# Asymmetric introgression between sympatric molestus and pipiens forms of *Culex pipiens *(Diptera: Culicidae) in the Comporta region, Portugal

**DOI:** 10.1186/1471-2148-9-262

**Published:** 2009-11-06

**Authors:** Bruno Gomes, Carla A Sousa, Maria T Novo, Ferdinando B Freitas, Ricardo Alves, Ana R Côrte-Real, Patrícia Salgueiro, Martin J Donnelly, António PG Almeida, João Pinto

**Affiliations:** 1 Centro de Malária e outras Doenças Tropicais, Instituto de Higiene e Medicina Tropical, Universidade Nova de Lisboa, Rua da Junqueira 96, 1349-008 Lisboa, Portugal; 2 Unidade de Entomologia Médica/Unidade de Parasitologia e Microbiologia Médicas, Instituto de Higiene e Medicina Tropical, Universidade Nova de Lisboa, Rua da Junqueira 96, 1349-008 Lisboa, Portugal; 3 Vector Group, Liverpool School of Tropical Medicine, Pembroke Place L3 5QA, Liverpool, UK

## Abstract

**Background:**

*Culex pipiens *L. is the most widespread mosquito vector in temperate regions. This species consists of two forms, denoted molestus and pipiens, that exhibit important behavioural and physiological differences. The evolutionary relationships and taxonomic status of these forms remain unclear. In northern European latitudes molestus and pipiens populations occupy different habitats (underground *vs*. aboveground), a separation that most likely promotes genetic isolation between forms. However, the same does not hold in southern Europe where both forms occur aboveground in sympatry. In these southern habitats, the extent of hybridisation and its impact on the extent of genetic divergence between forms under sympatric conditions has not been clarified. For this purpose, we have used phenotypic and genetic data to characterise *Cx. pipiens *collected aboveground in Portugal. Our aims were to determine levels of genetic differentiation and the degree of hybridisation between forms occurring in sympatry, and to relate these with both evolutionary and epidemiological tenets of this biological group.

**Results:**

Autogeny and stenogamy was evaluated in the F1 progeny of 145 individual *Cx. pipiens *females. Bayesian clustering analysis based on the genotypes of 13 microsatellites revealed two distinct genetic clusters that were highly correlated with the alternative traits that define pipiens and molestus. Admixture analysis yielded hybrid rate estimates of 8-10%. Higher proportions of admixture were observed in pipiens individuals suggesting that more molestus genes are being introgressed into the pipiens form than the opposite.

**Conclusion:**

Both physiological/behavioural and genetic data provide evidence for the sympatric occurrence of molestus and pipiens forms of *Cx. pipiens *in the study area. In spite of the significant genetic differentiation between forms, hybridisation occurs at considerable levels. The observed pattern of asymmetric introgression probably relates to the different mating strategies adopted by each form. Furthermore, the differential introgression of molestus genes into the pipiens form may induce a more opportunistic biting behaviour in the latter thus potentiating its capacity to act as a bridge-vector for the transmission of arboviral infections.

## Background

The *Culex pipiens *complex includes two of the most ubiquitous mosquito species in the world, *Culex quinquefasciatus *Say, 1823 in tropical and subtropical regions, and *Culex pipiens *L., 1758 in temperate regions. The nominal species of the complex, *Cx. pipiens *s.s., comprises two distinct forms, denoted pipiens and molestus, that are morphologically indistinguishable but exhibit important behavioural and physiological differences. The molestus form is stenogamous (mates in confined spaces, *i.e*. < 0.1 m^3 ^[1]), autogenous (can oviposit without a blood meal), mammophilic (prefers to feed on mammals, including humans) and homodynamic (remains active during winter). In contrast, the pipiens form is eurygamous (mates in open spaces), anautogenous (oviposition requires a blood meal), ornithophilic (feeds predominantly on birds) and heterodynamic (undergoes winter diapause) [2,3]. In the northern regions of Europe, Russia and USA, molestus and pipiens forms occupy different habitats, underground and aboveground, respectively [4-6].

The taxonomic status and evolutionary relationships of these forms remain controversial. One hypothesis is that the molestus form derives from surface pipiens populations that have undergone local adaptation to underground conditions [4]. Another hypothesis is that these forms may represent two distinct genetic entities [7]. Under the latter scenario, underground populations from northern Europe would have derived from southern autogenous populations that have subsequently dispersed and colonised underground habitats [7,8]. If in northern regions a physical discontinuity (underground vs. surface) is likely to significantly reduce gene flow between molestus and pipiens, hence promoting genetic isolation, the same may not hold for southern regions, where both autogenous and anautogenous populations co-occur in surface habitats [2,3,9]. Moreover, individuals with hybrid genetic signatures between molestus and pipiens have been described both in the USA and in southern Europe [6,7,10]. These results agree with reports of hybridisation between forms that result in hybrid females with intermediate physiological and behavioural traits [9,11]. Hybrids between molestus and pipiens forms are considered of great epidemiological importance. They can readily feed on both avian and mammalian hosts, including humans. This opportunistic biting behaviour will potentiate the role of *Cx. pipiens *as a bridge-vector for the transmission of arboviruses such as West Nile Virus (WNV), from their amplification hosts (birds) to humans [7,12].

Despite the conspicuous behavioural and physiological differences between molestus and pipiens, analysis of molecular markers revealed overall shallow genetic divergence and a paucity of diagnostic fixed differences between forms [8,13]. Exceptions are the contrasting differences in the degree of polymorphism found in the SH60 locus, a *Cx. pipiens *specific fragment originally described by Crabtree and co-workers [14] to distinguish this species from its tropical sibling *Cx. quinquefasciatus*, and the significant differentiation detected by analysis of microsatellites [7,8]. The most promising diagnostic marker so far obtained is a sequence difference in the flanking region of microsatellite CQ11, hereafter termed CQ11FL, that allows PCR-based discrimination of molestus, pipiens and putative hybrids [15].

In Portugal, *Cx. pipiens *is the most widespread mosquito species, reaching the highest densities in coastal estuarine areas during summer [16]. Some of these areas are important sanctuaries for migratory birds and hence potential sites for the introduction of arbovirus [17]. In the summer of 2004, WNV was isolated from *Cx. pipiens *collected in the southern province of the Algarve, in a mosquito survey that followed the description of two cases of WNV fever acquired by Irish bird-watchers in the region [18,19]. In Portugal, autogenous/stenogamous *Cx. pipiens*, typical of the molestus form, have been described from the analysis of larvae collected in urban surface habitats [20]. However, there is currently no information on the extent of genetic isolation between molestus and pipiens forms when they co-occur sympatrically in southern European aboveground habitats.

In this study, we used the CQ11FL marker and microsatellite loci to analyse samples of *Culex pipiens *collected aboveground in the estuarine region of Comporta in order to: i) determine levels of differentiation between samples displaying behavioural and physiological characteristics of pipiens and molestus forms; ii) assess the degree of hybridisation between forms and relate this with the potential for arbovirus transmission in the area.

## Results

### Autogeny, stenogamy and molecular identification

A total of 145 F1 families were analysed in the insectary to determine autogeny and stenogamy (Table 1). Of these, 115 (79.3%) were able to lay a first egg batch without blood feeding, hence being considered autogenous. The great majority of autogenous families (109 out of the 115) laid the first egg batch within two days after the emergence of the last adult. In the remaining 30 families (20.7%), oviposition occurred only after blood feeding in 11 (36.7%) and no oviposition was seen in the other 19 (63.3%) during the 10 days of the experiment. For subsequent comparisons, these families were put together into a single group denoted as non-autogenous.

**Table 1:** Autogeny and insemination rates in *Culex pipiens* from Comporta, Portugal

	INS = 0%	0%< INS <100%	INS = 100%	Total
Autogenous	1 (0.9)	30 (26.1)	84 (73.0)	115
Non-autogenous	22 (73.3)	8 (26.7)	0 (0.0)	30

Total	23 (15.9)	38 (26.2)	84 (57.9)	145

INS: proportion of inseminated females in each family.

**Figure 1:**
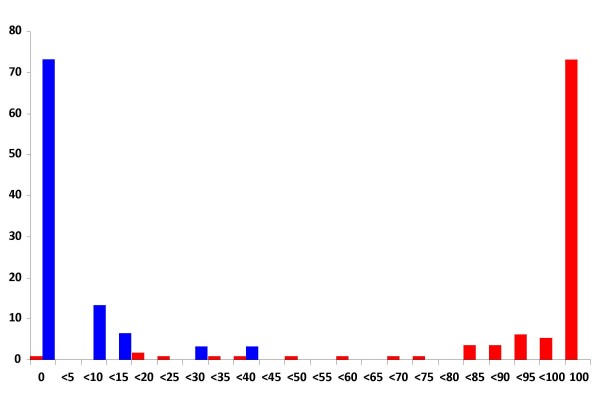
**Frequency distribution of insemination rates in autogenous and non-autogenous families of *Culex pipiens***. X-axis: proportion of inseminated females in each family at intervals of 5%. Y-axis: proportion of families (in percentage). Blue bars: non-autogenous; Red bars: autogenous.

There were significant associations of autogenous families with complete insemination and of non-autogenous families with absence of insemination (χ^2 ^= 100.7, d.f. = 2, *P *< 0.001; Table 1). In the autogenous group, the mean proportion of inseminated females was 92.9%, with 84 families (73.0%) showing 100% of inseminated females. There was a single autogenous family in which insemination was not observed. This family oviposited without blood feeding only after the two-days period from the emergence of the last adult, after which the family was subdivided (see Methods). In this family, the level of insemination could have been too low to accurately determining the insemination rate by observing the spermathecae, but also the possibility of a parthenogenic egg batch cannot be excluded [5]. In contrast, the non-autogenous group had a mean proportion of inseminated females of 4.1% and no inseminated females were observed in 22 (73.3%) families. The remaining 8 inseminated families all laid eggs but only after blood feeding. The frequency distribution of insemination rates was bimodal, with most of the observations concentrating in the extreme values (Figure 1). More than 91% of the autogenous families had insemination rates above 80% whereas over 93% of the non-autogenous families had insemination rates below 20%.

A total of 145 females were molecularly analysed, representing one female per family. Of these, 134 (92.4%) were identified as *Cx. pipiens *s.s. by *Ace2-*PCR [21]. For the remaining 11 females no amplified product was obtained despite several attempts changing PCR conditions, possibly due to alterations in the primers binding site. The families of these specimens were identified as belonging to *Cx. pipiens *s.s. by the observation of the genitalia of male siblings [22].

The genotypic frequencies of the CQ11FL marker are shown in Table 2. Overall, 78 (53.8%) females were homozygous for the 250 bp allele characteristic of the molestus form and 41 (28.3%) were homozygous for the 200 bp allele associated with the pipiens form. The remainder 26 (17.9%) females were heterozygous. There were significant associations between homozygous genotypes and alternative phenotypic traits. The "pipiens" genotype (CQ11FL_200/200_) predominated in non-autogenous and strictly non-stenogamous families (*i.e*. proportion of inseminated females = 0%) whereas the "molestus" genotype (CQ11FL_250/250_) was predominant in autogenous and strictly stenogamous families (*i.e*. proportion of inseminated females = 100%).

**Table 2:** Polymorphism at the flanking region of microsatellite CQ11 (CQ11FL) according to phenotypic groups of *Culex pipiens*.

	Total	Autogeny		Insemination rates		
			
		Autogenous	Non-autogenous	INS = 100%	0%< INS <100%	INS = 0%
CQ11FL_250/250_	78	77	1	60	17	1
	(53.8)	(67.0)	(3.3)	(71.4)	(44.7)	(4.3)
CQ11FL_200/250_	26	22	4	16	7	3
	(17.9)	(19.1)	(13.3)	(19.0)	(18.4)	(13.0)
CQ11FL_200/200_	41	16	25	8	14	19
	(28.3)	(13.9)	(83.3)	(9.5)	(36.8)	(82.6)
			
Chi-square tests		χ^2 ^= 58.9, d.f. = 2, *P *< 0.001		χ^2 ^= 51.7, d.f. = 4, *P *< 0.001		

INS: proportion of inseminated females in a family. Values in parenthesis refer to the relative genotypic frequencies (in percentage) within each phenotypic group. χ^2^: *P*-values of chi-square tests of homogeneity of genotypic frequencies among phenotypes.

### Microsatellite analysis

Genetic diversity estimates for the 14 microsatellite loci analysed are shown in Table S1, available in the Additional File 1. Apart from the whole sample (*N *= 145), calculations were also made for subsamples determined by genotypes at the CQ11FL locus. Although coincidence of genotypes and phenotypes was not absolute, the significant associations between CQ11FL homozygous genotypes and alternative phenotypes justified this tentative partitioning. Diversity estimates were lower in CQ11FL_250/250 _homozygotes (mean *A*_*R *_= 6, mean *H*_*e *_= 0.600) when compared to CQ11FL_200/200 _homozygotes (mean *A*_*R *_= 11, mean *H*_*e *_= 0.762). These differences were significant for both parameters (Wilcoxon signed-ranks tests; *A*_*R*_: *P = *0.001, *H*_*e*_: *P = *0.004). Microsatellite CQ11 was polymorphic in CQ11FL_200/200 _homozygous and in CQ11FL_200/250 _heterozygous groups. In contrast, this locus was nearly fixed for a 286 bp allele (*f = *0.984) in the CQ11FL_250/250 _homozygous group. This allele was also the most frequent in the heterozygous group (*f = *0.480) while it was absent in CQ11FL_200/200 _homozygotes.

Significant departures from Hardy-Weinberg proportions were detected in 10 loci (78.6%) when all specimens were analysed as a single sample (Table S1). Significant departures were seen at the same loci when analysis was repeated with pooled CQ11FL_250/250 _and CQ11FL_200/200 _homozygous specimens, *i.e*. when CQ11FL_200/250 _heterozygotes were excluded (data not shown). These departures were generally associated with significant positive *F*_*IS *_values indicative of a heterozygote deficit (Table S1). However, when the sample was subdivided according to CQ11FL genotypes, significant heterozygote deficits were observed only in seven occasions (16.7% out of 42 tests). Of these, locus CxpGT9 exhibited heterozygote deficits in all three subsamples, possibly reflecting locus-specific effects such as null alleles or selective pressures. There was also one significant departure that resulted from heterozygous excess, namely for locus CQ11 in the CQ11FL_200/250 _heterozygous group.

Exact tests of linkage disequilibrium revealed 62 (68.1%) significant associations between pairs of loci out of 91 tests performed for the whole sample. When each form was treated in separate, significant associations were reduced to 12 in the CQ11FL_250/250 _homozygous group, four in CQ11FL_200/200 _homozygous and one in CQ11FL_200/250 _heterozygous. Of the total 17 significant tests detected in the subsamples nine involved locus CxpGT9, that also showed significant heterozygote deficits. This locus was therefore excluded from subsequent analyses.

**Figure 2:**
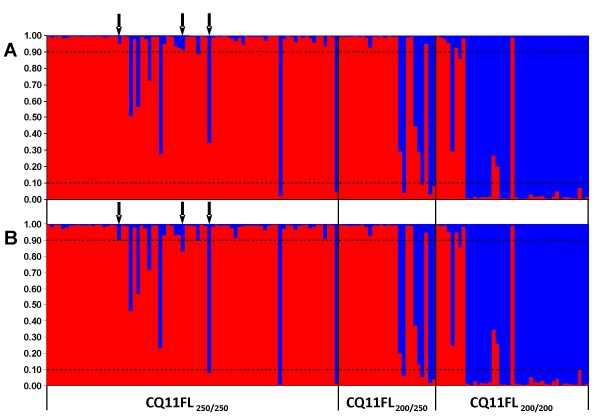
**Bayesian cluster analysis conducted by STRUCTURE **[23]. Columns correspond to the multilocus genotype of each individual, partitioned in two colours representing the probability of ancestry (*q*_*i*_) to each cluster. Red: cluster 1 (molestus); blue: cluster 2 (pipiens). Individuals were ordered according to their genotype at the CQ11FL locus. Dashed lines indicate the *q*_*i *_threshold used to determine admixed individuals (see Methods). A: analysis performed with 13 loci; B: analysis performed without locus CQ11. Arrows indicate individuals with different assignment between analyses.

Bayesian clustering analysis implemented by STRUCTURE [23] revealed two (*K = *2) genetically distinct ancestry clusters (Figure 2, A). Cluster 1 grouped 96 specimens, 70 (72.9%) of which had a homozygous CQ11FL_250/250 _genotype and seven (7.3%) were CQ11FL_200/200 _homozygotes. Interestingly, all 96 specimens assigned to cluster 1 belonged to autogenous families, with nearly 80% of these having 100% insemination rates and with all families displaying at least some proportion of inseminated females, thus providing support for cluster 1 to represent the molestus form (Table 3). In contrast, cluster 2 was representative of the pipiens form, with 30 (83.3%) out of the 36 specimens assigned presenting a CQ11FL_200/200 _homozygous genotype and only two (5.6%) were CQ11FL_250/250 _homozygotes. In this cluster, 75% of females belonged to non-autogenous families and nearly 65% were from families with no insemination. None of the females assigned to cluster 2 belonged to families with 100% insemination. Very similar results were obtained when microsatellite CQ11, which exhibited the highest allelic differences between CQ11FL genotypes, was removed from the analysis (Figure 2, B). With the exception of three individuals, all the remaining 142 (98%) specimens were assigned in to the same clusters as in the previous analysis, indicating that subdivision was not locus-dependent.

**Table 3:** Frequencies (in percentage) of genotypes at the CQ11FL locus and phenotypes for autogeny and insemination rates in each of the ancestry clusters revealed by STRUCTURE [23].

		CQ11FL genotype			Autogeny		Insemination rate		
				
	*N*	250/250	200/250	200/200	autogenic	non-autogenic	INS = 0%	0%<INS<100%	INS = 100%
Cluster 1	96	72.9	19.8	7.3	100.0	0.0	0.0	20.8	79.2
Admixed	13	46.2	23.1	30.7	76.9	23.1	0.0	38.5	61.5
Cluster 2	36	5.6	11.1	83.3	25.0	75.0	63.9	36.1	0.0

INS: proportion of inseminated females in a family.

There were 13 (9.0%) individuals of the total sample (*N *= 145) exhibiting an admixed ancestry (*i.e. q*_*i *_≥ 0.10 for both clusters). Of these, only 3 (23.1%) had a heterozygous CQ11FL_200/250 _genotype while the majority (76.9%) were homozygous for either of the two alleles present at the CQ11FL locus. Regarding phenotypes, the proportion of admixed individuals was lower in families that displayed alternative extreme traits (*i.e*. autogenous with 100% insemination and non-autogenous with no insemination: 8 out of 106 or 7.6%) when compared to the remaining families that were either autogenous or non-autogenous with a varying proportion of insemination above 0% and below 100% (5 out of 39 or 12.8%).

**Figure 3:**
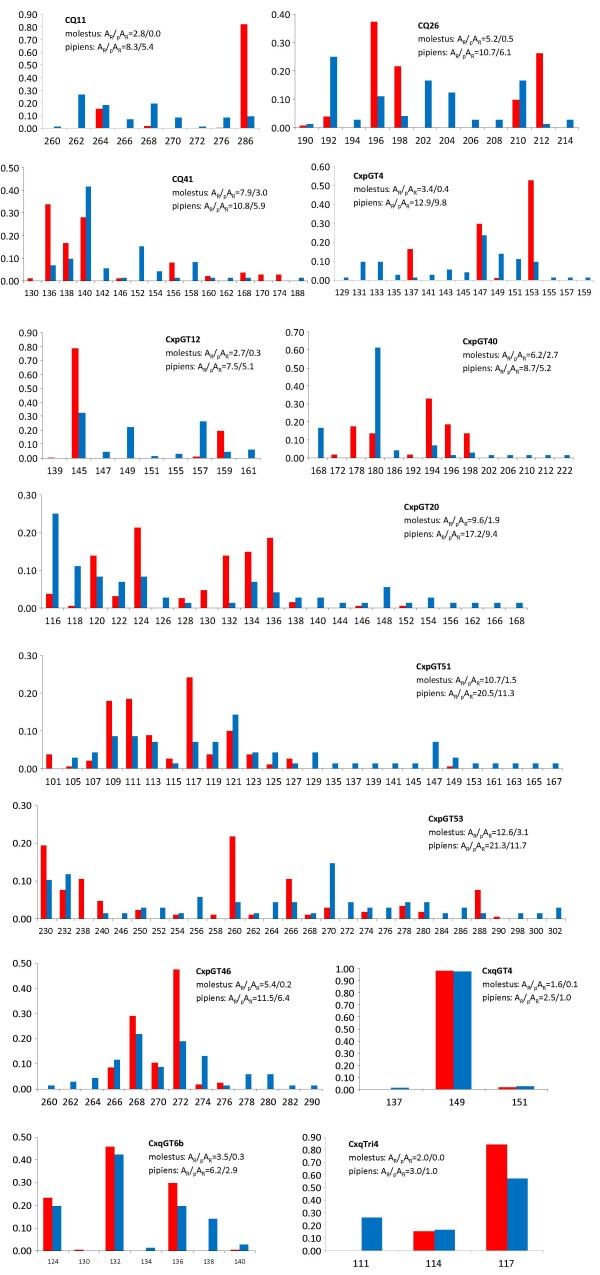
**Microsatellite allele richness and frequency in *Culex pipiens* of Comporta, Portugal**. Allele frequencies, allele richness (*A*_*R*_) and private alleles richness (_p_*A*_*R*_) were calculated for samples of common ancestry determined by Bayesian clustering analysis using STRUCTURE [23]. Red: molestus cluster, blue: pipiens cluster. X-axis: allele sizes in basepairs. Y-axis: allele frequency.

The microsatellite allele frequency arrays together with estimates of allele richness (*A*_*R*_) and private allele richness (_p_*A*_*R*_) for the clusters representative of the molestus and pipiens forms are shown in Figure 3. Allelic diversity was higher in the pipiens cluster, with a mean *A*_*R *_of 10 compared to a mean estimate of 6 for the molestus cluster. Most but not all of the alleles found in the molestus cluster were also represented in the pipiens cluster. In the molestus cluster _p_*A*_*R *_estimates per locus varied from 0 to 3 (mean = 1) whereas in the pipiens cluster _p_*A*_*R *_ranged from 1 to 12 (mean = 6). The pipiens and molestus clusters shared the most frequent allele at only four loci. For the remainder 9 loci, the most frequent alleles at each cluster were separated from each other on average by 8 basepairs, or four mutational steps (range: 2-12) as expected from their dinucleotide repeat constitution. The most remarkable difference was found in CQ11, with the most frequent alleles of pipiens and molestus being separated by 12 mutational steps.

Heterozygosity tests provided no evidence of recent population contraction in both molestus and pipiens clusters (Table 4). There was a single departure from Mutation Drift Equilibrium (MDE) in the pipiens cluster, that resulted from an apparent heterozygote deficiency (*P*_*He*<*Heq *_= 0.003) suggestive of population expansion and under the strict Stepwise Mutation Model (SMM).

**Table 4:** Results of heterozygosity tests [52] for molestus and pipiens clusters of *Cx. Pipiens*

		SMM	TPM (10%)	TPM (20%)	TPM (30%)
Cluster 1 (molestus)	*H*_*e*_>*H*_*eq*_	4	6	8	9
	*P*_*He *≠ *Heq*_	0.027	0.685	0.736	0.497
Cluster 2 (pipiens)	*H*_*e*_>*H*_*eq*_	2	3	6	8
	*P*_*He *≠ *Heq*_	**0.005**	0.057	0.340	0.893

*H*_*e*_>*H*_*eq*_: number of loci in which expected heterozygosity estimated from allele frequencies (*H*_*e*_) was higher than the estimate obtained from the number of alleles and sample size under MDE (*H*_*eq*_). *P*_*He *≠ *Heq*_: *P*-values of Wilcoxon tests to detect if *H*_*e *_differs from *H*_*eq *_in a significant number of loci. SMM: stepwise mutation model. TPM: two-phase model. In bold: significant *P*-value after correction for multiple testing by the sequential Bonferroni procedure.

A global *F*_*ST *_of 0.104 was obtained when subsamples were arranged according to the assignment into ancestry clusters revealed by STRUCTURE [23] (*i.e*. cluster 1, cluster 2 and admixed). The comparison between cluster 1 (molestus) and cluster 2 (pipiens) yielded a significant *F*_*ST *_of 0.127. Differentiation was generalised, in that significant *F*_*ST *_values were observed in 12 out of the 13 loci analysed, as shown in Table S2 of the Additional File 1. The single exception was locus CxqGT4, that was nearly monomorphic for the same allele in both forms (Figure 3). Locus CQ11 exhibited the highest *F*_*ST *_value (0.405) compared to the remaining loci (0.002-0.272). Excluding this locus from analysis resulted in a decrease of the overall *F*_*ST *_between molestus and pipiens to 0.103. Similar results were obtained with the *R*_*ST *_estimator (Table S2). In comparisons between molestus and pipiens, *R*_*ST *_was higher than *F*_*ST *_in 6 out of 13 loci and the mean over-loci estimates were also higher, with (*R*_*ST *_= 0.191) and without locus CQ11 (*R*_*ST *_= 0.123).

**Figure 4:**
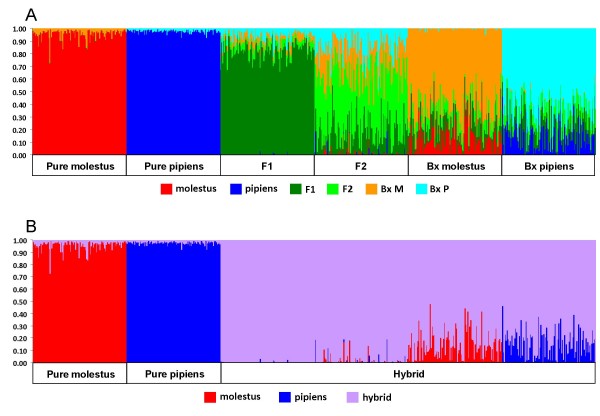
**Bayesian assignment of simulated purebred and hybrid individuals obtained by HYBRIDLAB **[25]**, as implemented by NEWHYBRIDS **[24]. Pure molestus, pure pipiens and hybrid (F1, F2 and backcrosses with each parental line) simulated individuals were generated from the genotypes of *Cx. pipiens *specimens that displayed by NEWHYBRIDS a *q*_*i*_>0.90 of being pure molestus (*N *= 100) and pure pipiens (*N *= 11). Simulations were done using HYBRIDLAB [25] to produce 100 simulated individuals for each class. Each vertical line represents a simulated individual. Lines are partitioned in colours according to the probabilities of assignment to each class. A: probabilities were obtained for each of the six classes. B: the "hybrid" category is the sum of probabilities of assignment to each of the four hybrid classes originally simulated.

The results of the admixture analysis performed by NEWHYBRIDS [24] on simulated genotypes generated by HYBRIDLAB [25] are shown in Figure 4 and in Table S3 of the Additional File 1. Maximum accuracy was achieved for all *Tq *but there were variations in power. All parental individuals were correctly identified at *Tq = *0.70 (minimum *q*_*i *_= 0.724). At this threshold, 93% of F1 hybrids were correctly assigned. Maximum power (*i.e*. 100% correct assignment) was obtained for this class at a *Tq *= 0.60. The analysis performed poorly in the assignment of the remaining hybrid classes, with proportions of correctly assigned individuals below 85% regardless of *Tq*. Given this poor performance, posterior probabilities of hybrid classes were summed and used as an estimate for the detection of hybrids but without definition of their admixture ancestry (Figure 4, B). For this category, maximum power was achieved only for *Tq *= 0.50. Based on these results, thresholds of 0.50 and 0.70 were used for the detection of hybrids on the real dataset.

**Figure 5:**
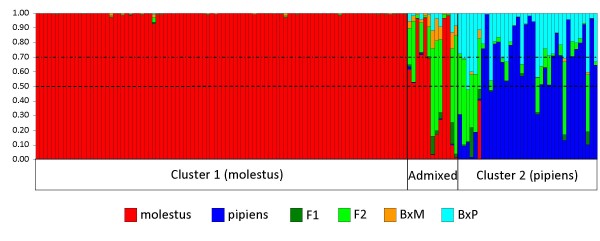
**Bayesian assignment of individuals into pure and hybrid classes as implemented by NEWHYBRIDS **[24]. Each column represents an individual analysed and it is partitioned into colours according to the probability of assignment to each of the six classes denoted in the label (pure molestus, pure pipiens, F1 hybrid, F2 hybrid, BxM: backcross with molestus, BxP: backcross with pipiens). Individuals were arranged according to their probability of ancestry obtained by STRUCTURE [23] analysis. Dashed lines highlight the two probability thresholds (*Tq*) used to assign individuals into classes (see Methods).

All individuals with a molestus ancestry (*N *= 96) revealed by STRUCTURE [23] were assigned to the same purebred class by NEWHYBRIDS [24] with probabilities of assignment close to 1 (minimum *q*_*i*_* = *0.927, Figure 5). In addition, five individuals of admixed ancestry were also included in this class. In contrast, of the 36 specimens with pipiens ancestry, only 26 (72.2%) displayed a *q*_*i*_ ≥ 0.50 of being assigned as parental pipiens (minimum *q*_*i*_* = *0.510). At *Tq = *0.70 this number decreased to 19 (52.8%) with a minimum *q*_*i*_* = *0.706. The individual probabilities of assignment into the parental pipiens class were lower than those of purebred molestus. For individuals assigned as parental pipiens, the average proportion of assignment into a different class (*i.e*. molestus and/or hybrid) was 0.144 for *Tq *= 0.70 and 0.218 for *Tq *= 0.50.

Depending on the threshold, the proportion of hybrid individuals detected by NEWHYBRIDS [24] varied between 7.6% (*Tq *= 0.70) and 10.3% (*Tq *= 0.50), values comparable to the 9.0% proportion obtained by STRUCTURE [23] analysis (Table 5).

**Table 5:** Proportions of pure and admixed *Culex pipiens* individuals inferred by Bayesian assignment methods implemented by SRUCTURE [23] and NEWHYBRIDS [24]

	molestus	admixed	pipiens
STRUCTURE	96 (66.2)	13 (9.0)	36 (24.8)
NEWHYBRIDS (*Tq *= 0.50)	104 (71.7)	15 (10.3)	26 (17.9)
NEWHYBRIDS (*Tq *= 0.70)*	101 (69.7)	11 (7.6)	19 (13.1)

* At this threshold, only 131 specimens were assigned to classes. The remainder 14 analysed individuals presented *qi*<0.70 of belonging to any of the classes and were thus undetermined.

## Discussion

Insectary experiments based on the progeny of field-caught *Cx. pipiens *females revealed strong associations between alternative traits that define molestus and pipiens forms. The highest proportions of inseminated females were seen in autogenous families. These two associated traits are expected for an autogenous/stenogamous molestus population. Conversely, non-autogenous families exhibited the lowest insemination rates suggesting that these families represent the anautogenous/eurygamic pipiens population. The non-autogenous group included families that oviposited after a blood meal and those in which no oviposition was detected throughout the experiment. Factors such as poor adaptation to insectary conditions causing gonotrophic dissociation could have resulted in the absence of oviposition in families that otherwise could in fact be autogenous. On the other hand, low insemination rates could also determine the lack of oviposition. Coincidently, no inseminated females were detected in all the 19 families that did not oviposit after blood feeding. Under the experimental conditions used, absence of insemination reflects the inability of mating in confined spaces, a trait of the pipiens form.

The observed phenotypic separation was confirmed by microsatellite analysis. Extensive heterozygote deficits and linkage between loci were detected when all individuals were treated as a single sample. These departures were greatly reduced when the sample was tentatively subdivided into subsamples defined by the CQ11FL locus, a single-locus marker available to distinguish molestus and pipiens forms [15]. The Bayesian method of Pritchard and co-workers [23] identifies clusters from multilocus genotypic frequencies based on the minimisation of departures from Hardy-Weinberg equilibrium and of linkage disequilibrium between loci. This analysis revealed two distinct genetic clusters that were largely coincident with the molestus and pipiens forms defined by both the phenotypic traits and the CQ11FL locus. Altogether, these results suggest that molestus and pipiens forms represent distinct gene pools of a subdivided *Cx. pipiens *population.

From the comparison with the ancestry groups revealed by STRUCTURE [23], CQ11FL was only partially effective as a diagnostic marker. There was a good concordance between alternative homozygous genotypes and each form but heterozygous CQ11FL genotypes performed less well in determining admixed individuals. Under conditions of continued hybridisation, recombination and independent assortment will break the linkage between alternative diagnostic genotypes and their respective genetic ancestry background. As pointed by Bahnck and Fonseca [15], results from this marker should thus be interpreted only at the population level. Nevertheless CQ11FL still served as a good indicator of the sympatric presence of both molestus and pipiens forms in the study area.

Based on the partitioning of samples according to ancestry clusters revealed by STRUCTURE [23], a global *F*_*ST *_of 0.127 was obtained between molestus and pipiens forms. This estimate is slightly lower but still comparable to those reported in previous comparisons between underground molestus and aboveground pipiens populations (usually between 0.130 and 0.190) using similar microsatellite datasets [6,26]. Although no molestus underground populations from the study area were available for comparison, it appears that gene flow between molestus and pipiens forms is not significantly increased by the sympatric co-existence of both populations in the surface. This argument plays in favour of the hypothesis of at least partial reproductive isolation between molestus and pipiens forms and that the under/aboveground physical discontinuity is not the only factor promoting genetic divergence, as previously debated [4,7,8]. Under this particular situation of sympatry, positive reinforcement may play a role in counteracting the effects of gene flow [27], hence maintaining isolation between forms.

Microsatellite CQ11 displayed the highest differentiation between molestus and pipiens, with an *F*_*ST *_estimate *ca*. 2-fold greater than for the other loci. This locus was close to fixation in molestus form for a 286 bp allele, but this was a low-frequency allele in the pipiens form (Figure 3). This allelic profile is not unique for the study area. High frequencies of a CQ11 allele in the same size range (283-285 bp) have been reported for underground and aboveground molestus populations from Europe and the USA [7,8,15]. This continental-wide genetic signature is consistent with a single evolutionary origin of the molestus form, possibly arising in the southern latitudes of Europe or North Africa as a human-adapted commensal form, that later dispersed into northern latitudes as underground suitable habitats became available [7]. Furthermore, this locus-specific differentiation may indicate that CQ11 locates in a genomic region under divergent selection. In these genomic regions, reduced recombination and selection against introgression maintain differentiation not only at loci associated with traits of ecological adaptation or reproductive isolation but also at surrounding neutral loci through genetic hitchhiking [28,29]. This mechanism is considered a major process of sympatric/ecological speciation and has been described in several insect species [30-32]. Genome-wide scans will be necessary to confirm the presence of such genomic regions in *Cx. pipiens*.

Estimates of hybrid rates between molestus and pipiens forms between 7-10% were obtained by STRUCTURE [23] and NEWHYBRIDS [24] admixture analysis. These values are similar to the estimates obtained for southern European aboveground populations (10%) using STRUCTURE [23], although the authors used a different *Tq *of 0.06 [7]. Adjusting ancestry assignment to this threshold still yielded a comparable hybrid rate of 15.2% for our sample. In comparisons between underground molestus and aboveground pipiens populations from the USA hybrid rates of 12% have been documented [6] but up to 40% admixed individuals have been documented in USA *Cx. pipiens *populations by Fonseca and co-workers [7]. According to the authors, a more recent colonisation and posterior contact of separate Old World molestus and pipiens populations may explain the higher levels of hybridisation found in the USA. On the other hand, the low levels of hybridisation in southern European *Cx. pipiens *populations, even when both forms occur sympatrically as here demonstrated, provides additional support for reproductive/ecological barriers to gene flow other than habitat segregation.

The degree of microsatellite differentiation in our dataset was insufficient to identify hybrids beyond the F1 class, as revealed by the analysis of simulated data. This was not an unexpected result as NEWHYBRIDS [24] often requires a large number of highly diagnostic markers between populations to identify F2 and backcrossed hybrids with confidence [33,34]. However, this analysis revealed important differences in the proportions of admixture within forms. Individuals with molestus ancestry were all classified as purebred molestus with probabilities of assignment above 0.92. In contrast, individuals with pipiens ancestry had a mean proportion of admixture of 0.387 (as measured by the individual posterior probabilities of belonging into a non-pipiens class) and 28-48% (depending on *Tq*) were classified as hybrids. These differences suggest a pattern of asymmetrical gene flow, in which higher proportions of molestus alleles are introgressed into the pipiens form. A similar trend has also been described in a population from Chicago IL (USA), in which the pipiens form presented higher proportions of molestus and *Cx. quinquefasciatus *ancestry [26].

Another hypothesis could be raised if the molestus form would have locally evolved from the pipiens form through a recent founding event. Under this scenario, the microsatellite composition of the molestus population would be made almost exclusively of only a subset of the alleles present in the pipiens form which might result in an apparent signal of admixture in the latter. While estimates of allele and private allele richness seem to support this view, there were considerable differences between forms in the microsatellite allele arrays that are not consistent with this hypothesis. These differences are illustrated by the number of mutational steps separating the most frequent alleles at each locus. Size variance-based *R*_*ST *_values were higher than frequency-based *F*_*ST *_values in nearly half of the loci and also for the mean over-loci estimates. Higher *R*_*ST *_estimates do not conciliate with a recent founding event that would otherwise imply that genetic drift rather than mutation would be the primary evolutionary force shaping genetic divergence between forms [35]. Moreover, heterozygosity tests provided no evidence for the molestus form to have recently undergone any major population reduction that would be expected from a founding event. Finally, the peculiar composition of the CQ11 microsatellite in the molestus form, displaying a high frequency allele common to all other molestus populations regardless of geographic origin is also not consistent with local multiple origins of the molestus form. Altogether, these evidences render the hypothesis of the molestus population being derived from the local pipiens form unlikely. Extending the analysis to other regions of sympatry between molestus and pipiens would provide insights on whether the observed patterns of introgression are a local phenomenon or a general trend for the species in its southern distribution.

The mechanisms underlying the patterns of asymmetrical introgression between molestus and pipiens are unknown. One hypothesis can be drawn from the different mating strategies displayed by molestus and pipiens forms. Preferential introgression from molestus to pipiens could be expected if stenogamous molestus males mate readily with both molestus and pipiens females in aboveground habitats. On the other hand, pipiens males require open spaces to mate due to swarm-based mating behaviour [36]. This more specialised behaviour may result in a higher propensity to mate with pipiens females. This hypothesis relies on two main assumptions. The first is that introgression between molestus and pipiens is mainly male-mediated and to test for this hypothesis the analysis of sex-linked markers would be required. In a recent study analysing Asian populations of two additional members of the *Cx. pipiens *complex, the allele specific of *Cx. quinquefasciatus *at the sex-linked *Ace-2 *locus was found to have introgressed into *Culex pipiens pallens *Coquillett, 1898 through the males [37]. Patterns of male-mediated asymmetrical introgression have also been reported in several other non-insect organisms, such as tree frogs [38], warbler birds [39], mouse lemurs [40] and macaque monkeys [41]. The second assumption is that both pipiens and hybrid females have a greater propensity for seeking swarms for mating. To address this question, more studies are needed to characterise the swarming and mating behaviours in *Cx. pipiens*, in areas of sympatry between forms.

The molestus form was predominant in the study area and this trend appeared to be maintained throughout the year (data not shown). While this factor may also contribute to a higher introgression of genes from molestus to pipiens, it may also suggest fitness differences between forms. In southern regions with mild winters, the inability of the molestus form to undergo diapause during winter may be a lesser disadvantage than at northern latitudes. When occurring in sympatry with the pipiens form in surface habitats, autogeny and a more generalist mating behaviour are likely to result in a greater fitness molestus form.

## Conclusion

Both physiological/behavioural and genetic data provide evidence for the sympatric occurrence of molestus and pipiens forms of *Cx. pipiens *in aboveground habitats of the study area. In spite of the sympatric occurrence, estimated hybridisation rates were not much higher than those reported in ecological settings where both forms are physically separated which suggests at least partial reproductive isolation between molestus and pipiens. More importantly, hybridisation appears not to be bidirectional and this is possibly a result of the different mating strategies exhibited by each form. The observed patterns of asymmetrical introgression may have epidemiological repercussions. In two recent studies covering three USA States, pipiens form females that have fed upon mammals (humans in particular) presented significantly higher proportions of molestus genetic ancestry [10,26]. These findings suggest a genetic basis for host selection by *Cx. pipiens*. The introgression of molestus genes into the pipiens form may induce a more opportunistic biting behaviour thus potentiating the capacity of the latter form to act as a bridge-vector for the transmission of arbovirus such as WNV [12]. Further studies focusing on the feeding habits and population dynamics of molestus and pipiens forms are required in order to clarify the impact of hybridisation in the vectorial capacity of *Cx. pipiens *and, consequently, on the potential for transmission of arboviral infections.

## Methods

### Study region and mosquito collection

Mosquito collections took place between May 2005 and August 2006 in the Comporta region (38° 22' 60 N, 8° 46' 60 W), District of Setubal, Portugal. Comporta is a low-lying area (altitude <60 m) with diverse ecotypes. Residential areas are situated along a national road that crosses the study region from north to south. The south and east is mainly occupied by pine forest (*Pinus pinaster *Aiton, 1789; *Pinus pinea *L., 1753) and semi-natural agro-forest systems of cork-oak (*Quercus suber *L., 1753). In the west there are extensive areas of rice fields and a system of sand-dunes. The north and northwest is part of a protected landscape area occupied by marshland, rice fields and saltpans. This protected area extends northwards into the national wildlife reserve of Estuário do Sado. The reserve harbours over 240 bird species. These include migratory birds such as the European starling (*Sturnus vulgaris *L., 1758), the mallard (*Anas platyrhynchos *L., 1758) and the white stork (*Ciconia ciconia*, L. 1758), that have been reported as WNV hosts [17].

The region has a warm temperate climate with a dry hot summer and a mild winter (class Csa, Köppen Classification System [42]). Monthly averages of mean daily temperatures vary between 10°C and 21°C and relative humidity between 76% and 89%. Monthly averages of daily rainfall fluctuate between 0.12 and 3.4 mm.

Bimonthly mosquito collections were made by indoors resting captures and CDC light traps baited with CO_2 _inside animal shelters (chicken, rabbit and pig). Collected live mosquitoes were transported to the laboratory and identified to species or complex of sibling species using morphological keys [22].

### Determination of autogeny and stenogamy

Blood fed and gravid *Cx. pipiens *females were placed in individual cages in an Insectary (25 ± 2°C; 70 ± 10% RH) until oviposition. Individual egg rafts were reared until the adult stage to obtain F1 families. Pupae from each F1 family were transferred into cages with 20 cm side (0.008 m^3^) for adult emergence. After emergence of the first adult the family was kept in the cage with access to a fructose 10% solution and an oviposition tray. Both pupae and oviposition trays where daily observed for the presence of egg-rafts. If oviposition occurred until two days after the emergence of the last adult (*i.e*. on average 14 days after the emergence of the first adult of the egg batch) the family was deemed autogenous. Families that did not lay eggs during this period were divided into two cages keeping similar sex ratios in each cage. In one of the cages mosquitoes were maintained in similar conditions as previously in order to recover eventually autogenous families that had delayed oviposition. In the other cage, females were given the opportunity to take a daily blood feed on a vertebrate host (mouse and chicken) for a period of 10 days.

After the end of the experiment, all F1 specimens were sacrificed by chilling. Females had their abdomen dissected to determine if their spermatheca was inseminated, as an indicator of the capacity to mate in confined spaces. The head and thorax of each female were preserved in individual tubes with silica gel and kept at room temperature until DNA extraction.

### Molecular analyses

DNA extraction from individual F1 females was performed by the method of Collins and co-workers [43]. Specimens were identified to species of the *Culex pipiens *complex by a multiplex PCR assay that targets species-specific polymorphisms at the intron-2 of the acetylcholinesterase-2 gene (*Ace-2*), using primers specific for *Cx. pipiens *s.s., *Culex torrentium *Martini, 1925 and *Cx. quinquefasciatus *[21]. The first two species have been annotated for Portugal [22]. Although *Cx. quinquefasciatus *has not been found in Portugal, its subtropical distribution with a northern limit around 36° latitude prompted us to test this additional primer. The PCR assay described by Bahnck & Fonseca [15] was used to detect a size polymorphism in the 5' flanking region of the CQ11 microsatellite of *Cx. pipiens*. This marker, here denoted as CQ11FL, differentiates specimens of the pipiens form, that display a PCR product of 200 bp, from the molestus form (250 bp). Hybrids exhibit both amplicons (200 bp/250 bp).

Fourteen microsatellite loci [44-46] were analysed in this study (Table S4, Additional File 1). Each locus was amplified separately in a 20 μl PCR reaction that contained 1× GoTaq^® ^Flexi Buffer (Promega, USA), 2.5 mM MgCl_2_, 0.20 mg/ml Bovine Serum Albumin, 0.25 mM dNTPs, 0.20 μM of each primer and 0.5 U GoTaq^® ^Flexi DNA polymerase (Promega, USA). For each locus, one of the primers was fluorescently labelled (NED, HEX or 6-FAM; Applied Biosystems, USA). Thermocycling conditions included an initial denaturation step of 5 min at 96°C followed by 30 cycles each with 96°C for 30 s, Annealing at 52°C-58°C (locus dependent, Table S4) for 30 s, and 72°C for 30 s. After a final extension step of 5 min at 72°C, reactions were stopped at 4°C.

Amplified products were separated by capillary electrophoresis in a genetic analyser ABI3730 (Applied Biosystems, USA) at the DNA Analysis Facility on Science Hill, Yale University (USA). Fragment sizes and genotypes were scored using the software GeneMarker 1.4. (Softgenetics, USA).

### Data analysis

Pearson's Chi-square tests were used to determine associations between autogeny and stenogamy phenotypic traits and with CQ11FL genotypes.

Genetic diversity at each microsatellite locus was characterised by estimates of expected heterozygosity using Nei's unbiased estimator [47] and inbreeding coefficient (*F*_*IS*_). Significance of *F*_*IS *_values was assessed by randomisation tests. These analyses were performed using FSTAT v. 2.9.3.2. [48]. In addition, estimates of allele richness (*A*_*R*_) and private allele richness (_p_*A*_*R*_) adjusted for the lowest sample size were obtained by a rarefaction statistical approach implemented by the programme HP-RARE [49].

Departures from Hardy-Weinberg proportions were tested by exact tests available in ARLEQUIN v.3.11 [50]. The same software was used to perform exact tests of linkage disequilibrium between pairs of loci based on the expectation-maximisation approach described by Slatkin and Excoffier [51]. Cornuet and Luikart's [52] heterozygosity tests were used to detect recent population perturbations. This method compares two estimates of expected heterozygosity, based on allele frequency (*H*_*e*_) and on the number of alleles and sample size (*H*_*eq*_), respectively. At mutation-drift equilibrium (MDE), both estimates should be similar but if a population experiences a recent bottleneck there will be a transient state in which *H*_*e*_*>H*_*eq *_due to a rapid loss of rare alleles. Conversely *H*_*e*_*<H*_*eq *_is an indicator of a recent population expansion. Estimates of *H*_*eq *_under MDE were obtained assuming a strict stepwise mutation model (SMM) and two-phase models (TPM) with proportions of indels larger than one repeat of 10%, 20% and 30%. Wilcoxon tests were used to determine if there were a significant number of loci in which *H*_*e *_≠ *H*_*eq *_as an indication of departure from MDE. Calculations were done using BOTTLENECK version 1.2.02 [52].

Genetic differentiation between groups was measured by estimates of the fixation index, *F*_*ST*_, calculated according to Weir and Cockerham [53]. Genotypic permutation tests available in FSTAT [48] were performed to infer if the estimates differed significantly from zero. The microsatellites equivalent *R*_*ST *_[35] was estimated as implemented by ARLEQUIN [50].

Bayesian clustering analysis as implemented by STRUCTURE 2.2 [23] was used to infer population substructure/ancestry from the dataset without prior information of sampling groups (*i.e*. phenotypes), under the admixture model with correlated allele frequencies. Ten independent runs with 10^5 ^burn-in steps and 10^5 ^iterations were done for each value of *K *(*K *= 1 to 4 clusters). The method of Evanno and co-workers [54] was used to determine the most likely number of clusters in the sample. Following the suggestions of Vaha and Primmer [34], individual genetic assignment to clusters was based on a minimum posterior probability threshold (*Tq*) of 0.90. Individuals displaying 0.1 ≤ *q*_*i *_≤ 0.90 were considered of admixed ancestry.

The Bayesian method implemented by NEWHYBRIDS 1.1. [24] was used to assign individuals into 6 classes: pure molestus, pure pipiens, and hybrids (F1, F2 and backcrosses with molestus or pipiens). The approach of uniform priors was used and results were based on the average of five independent runs each with 10^5 ^burn-in steps and 10^5 ^iterations.

The performance of NEWHYBRIDS to detect purebred and hybrid individuals with the present microsatellite dataset was assessed using simulated data generated by HYBRIDLAB [25]. From the initial NEWHYBRIDS analysis, pure molestus and pipiens individuals were selected based on a *q*_*i*_>0.90. From this sampling, 100 simulated genotypes of each parental and hybrid class were generated. These artificial genotypes, without prior population information, were analysed in NEWHYBRIDS. Following the examples of previous works [34,55], power (number of correctly identified individuals for a class over the actual number of individuals of that class) and accuracy (number of correctly identified individuals for a class over the total number of individuals assigned to that class) were calculated for four *Tq *values (0.50, 0.70, 0.80 and 0.90). Analysis was based on the mean of five replicates of simulated datasets.

Whenever multiple testing was performed, the nominal significance level of rejection of the null hypothesis (α = 0.05) was corrected by the sequential Bonferroni procedure [56].

## Authors' contributions

BG, MTN, CAS, FBF, RA and APGA carried out sample collections and insectary experiments. Molecular analyses were conducted by BG, PS, ARCR and FBF. BG, PS, ARCR and JP performed the genetic data analysis. MTN, CAS, APGA, MJD and JP conceived the study and designed the experiments. BG and JP drafted the manuscript with the contributions of PS, FBF, RA and MJD. All authors read and approved the final manuscript.

## Supplementary Material

Additional file 1 **Tables S1, S2, S3 and S4**. Table S1. Genetic diversity at microsatellite loci of *Culex pipiens *from Portugal. Table S2. Estimates of *F*_*ST *_and *R*_*ST *_between forms of *Culex pipiens *identified by Bayesian clustering analysis performed in STRUCTURE [23]. Table S3. Power and accuracy of NEWHYBRIDS to detect purebred and hybrid simulated individuals. Table S4. Microsatellite loci analysed. Click here for file
