# Supplementary material for: Asymmetric introgression between sympatric molestus and pipiens forms of Culex pipiens (Diptera: Culicidae) in the Comporta region, Portugal
Source: BMC Evol Biol. 2009 Nov 6;9:262. doi: 10.1186/1471-2148-9-262 (PMC2778655; doi:10.1186/1471-2148-9-262)
Supplement: Additional file 1 — Tables S1, S2, S3 and S4. Table S1. Genetic diversity at microsatellite loci of Culex pipiens from Portugal. Table S2. Estimates of FST and RST between forms of Culex pipiens identified by Bayesian clustering analysis performed in STRUCTURE [23]. Table S3. Power and accuracy of NEWHYBRIDS to detect purebred and hybrid simulated individuals. Table S4. Microsatellite loci analysed. [file 1471-2148-9-262-S1.pdf]

Additional file 1

Table S1 - Genetic diversity at microsatellite loci of *Culex pipiens* from Portugal

Locus		$CQ11FL_{250/250}$ (N=78)	$CQ11FL_{200/250}$ (N=26)	$CQ11FL_{200/200}$ (N=41)	All samples (N=145)
CQ11	A_R	1.7	4.8	7.2	6.5
	H_e	0.031	**0.620**	0.801	**0.607**
	F_{IS}	-0.004	**-0.548***	0.190	**0.346**
CQ26	A_R	6.6	7.0	10.1	8.7
	H_e	**0.756**	**0.826**	0.862	**0.835**
	F_{IS}	**0.537**	**0.579**	0.001	**0.396**
CQ41	A_R	9.3	8.8	12.0	10.8
	H_e	0.789	0.789	**0.833**	**0.812**
	F_{IS}	0.144	0.088	**0.262**	**0.179**
CxpGT04	A_R	4.4	8.5	11.9	9.0
	H_e	0.637	0.727	0.879	**0.748**
	F_{IS}	0.092	-0.111	0.062	0.077
CxpGT09	A_R	6.1	10.0	10.3	9.3
	H_e	**0.743**	**0.855**	**0.847**	**0.818**
	F_{IS}	**0.521**	**0.491**	**0.366**	**0.482**
CxpGT12	A_R	3.9	4.9	7.4	5.9
	H_e	0.380	0.470	0.766	**0.545**
	F_{IS}	0.184	0.202	0.106	**0.213**
CxpGT20	A_R	10.8	12.5	16.7	13.2
	H_e	**0.867**	0.888	0.916	**0.895**
	F_{IS}	0.135	-0.082	0.121	**0.104**
CxpGT40	A_R	6.6	6.8	9.6	8.1
	H_e	0.805	0.735	0.719	**0.814**
	F_{IS}	0.038	0.238	0.185	**0.166**
CxpGT46	A_R	5.5	8.8	10.1	8.1
	H_e	0.667	0.776	0.853	**0.748**
	F_{IS}	0.110	0.034	0.194	**0.131**
CxpGT51	A_R	11.8	13.4	18.3	14.3
	H_e	0.866	0.885	0.932	0.891
	F_{IS}	0.131	0.044	0.035	0.089
CxpGT53	A_R	14.1	12.7	22.4	16.5
	H_e	**0.893**	0.888	0.954	**0.914**
	F_{IS}	**0.186**	0.009	0.106	**0.134**
CxqQGT4	A_R	1.7	2.8	1.8	1.8
	H_e	0.038	0.076	0.048	0.047
	F_{IS}	-0.013	-0.010	-0.013	-0.018
CxqGT6B	A_R	3.3	3.9	6.9	5.0
	H_e	0.635	0.686	0.754	0.681
	F_{IS}	0.079	-0.166	0.006	0.017
CxqTRI4	A_R	2.5	3.0	3.6	3.1
	H_e	0.301	0.306	0.543	0.380
	F_{IS}	0.105	0.121	0.146	0.148
All loci	A_R	6.3	7.7	10.6	8.6
	H_e	**0.601**	**0.681**	**0.765**	**0.695**
	F_{IS}	**0.192**	**0.078**	**0.135**	**0.190**

N: sample size. A_R: allelic richness; H_e: expected heterozygosity; F_{IS}: inbreeding coefficient. Values in bold indicate a significant P-value after correction for multiple tests (see Methods). *Significant P-value for a negative F_{IS}. Per locus and over samples Hardy-Weinberg tests were performed using ARLEQUIN [50]. For over loci estimates the global test available in FSTAT [48] was used.

Table S2 - Estimates of F_{ST} and R_{ST} between forms of *Culex pipiens* identified by Bayesian clustering analysis performed in STRUCTURE [23]

Locus		Cluster 1 (molestus) vs. Admixed	Admixed vs. Cluster 2 (pipiens)	Cluster 1 (molestus) vs. Cluster 2 (pipiens)	All samples
CQ11	F_{ST}	**0.207**	0.032	**0.405**	**0.360**
	R_{ST}	**0.266**	**0.239**	**0.612**	**0.566**
CQ26	F_{ST}	0.099	-0.017	**0.132**	**0.117**
	R_{ST}	0.079	0.018	0.004	0.017
CQ41	F_{ST}	0.038	0.014	**0.072**	**0.059**
	R_{ST}	0.059	-0.013	0.025	0.030
CxpGT4	F_{ST}	**0.043**	0.033	**0.155**	**0.120**
	R_{ST}	0.032	0.001	**0.151**	**0.110**
CxpGT12	F_{ST}	**0.072**	0.049	**0.272**	**0.216**
	R_{ST}	-0.020	0.061	**0.131**	**0.099**
CxpGT20	F_{ST}	**0.023**	0.045	**0.060**	**0.050**
	R_{ST}	0.082	0.013	-0.009	0.009
CxpGT40	F_{ST}	-0.019	**0.184**	**0.205**	**0.164**
	R_{ST}	-0.022	**0.157**	**0.267**	**0.219**
CxpGT46	F_{ST}	-0.043	0.061	**0.060**	**0.049**
	R_{ST}	-0.043	-0.058	0.010	0.001
CxpGT51	F_{ST}	0.002	0.021	**0.025**	**0.019**
	R_{ST}	**0.151**	0.063	**0.346**	**0.284**
CxpGT53	F_{ST}	0.020	0.010	**0.035**	**0.032**
	R_{ST}	0.123	-0.038	**0.076**	**0.078**
CxqGT4	F_{ST}	-0.007	-0.024	0.002	-0.004
	R_{ST}	-0.008	-0.015	0.007	0.001
CxqGT6b	F_{ST}	-0.003	0.001	**0.015**	**0.010**
	R_{ST}	0.033	0.093	0.010	0.024
CxqTri4	F_{ST}	-0.009	0.053	**0.164**	**0.119**
	R_{ST}	-0.018	**0.146**	**0.321**	**0.256**
All loci	F_{ST}	**0.031**	**0.039**	**0.127**	**0.104**
	R_{ST}	-0.003	-0.055	**0.191**	**0.135**
Without CQ11	F_{ST}	**0.021**	**0.039**	**0.103**	**0.082**
	R_{ST}	-0.016	-0.067	**0.123**	**0.079**

Individuals with a minimum posterior probability $0.1 \leq q_i \leq 0.90$ were considered admixed genotypes between the two clusters (molestus and pipiens). In bold: significant F_{ST} or R_{ST} after correction for multiple testing by the sequential Bonferroni procedure [56].

Table S3. Power and accuracy of NEWHYBRIDS to detect purebred and hybrid simulated individuals

Class	Tq = 0.900		Tq = 0.800		Tq = 0.700		Tq = 0.600		Tq = 0.500	
	Power	Accuracy	Power	Accuracy	Power	Accuracy	Power	Accuracy	Power	Accuracy
Pure molestus	0.890	1.000	0.990	1.000	1.000	1.000	1.000	1.000	1.000	1.000
Pure pipiens	1.000	1.000	1.000	1.000	1.000	1.000	1.000	1.000	1.000	1.000
Hybrid	0.678	1.000	0.860	1.000	0.953	1.000	0.988	1.000	1.000	1.000
F1	0.110	1.000	0.720	1.000	0.930	1.000	1.000	1.000	1.000	1.000
F2	0.000	-	0.010	1.000	0.030	1.000	0.160	1.000	0.410	1.000
Bx molestus	0.010	1.000	0.110	1.000	0.410	1.000	0.620	1.000	0.840	1.000
Bx pipiens	0.000	-	0.060	1.000	0.280	1.000	0.570	1.000	0.830	1.000

Five threshold values (Tq) were analysed for power and accuracy to detect parental and hybrid simulated individuals. The "hybrid" category represents the sum of assignment probabilities to each of the four hybrid lineages originally tested. Power: number of correctly identified individuals for a class over the actual number of individuals of that class in the sample (N=100 for purebred, F1, F2, Bx molestus and Bx pipiens; N=400 for the hybrid category). Accuracy: number of correctly identified individuals for a class over the total number of individuals assigned to that class.

Table S4. Microsatellite loci analysed

Locus	Repeat	Primers	T_A (ºC)	Reference
CQ11	$(GT)_2ACTTC(GT)_9$	F: GATCCTAGCAAGCGAGAAC<br>R: 6-fam-GAGCGGCCAAATATTGAGAC	52	Fonseca *et al.* 1998
CQ26	$(GTGTGTAT)_2+(GT)_{10}+(GT)_5$	F: TCCGACATGGGAAGAGCGCA<br>R: 6-fam-ACGCGCCCTTCTTCTGCAAC	56	Fonseca *et al.* 1998
CQ41	$(GT)_{12}$	F: CTGCCACTGCCTGACTGAAA<br>R: Hex-ACCACTCAGCAACATCCGGC	52	Fonseca *et al.* 1998
CxpGT4	$(GT)_5(GTTT)_2GC(GT)_2CT(GT)_5$	F: GTCGTCGCTAACCCTTGTT<br>R: Ned-CGCGATAGTCGGTAATCGT	54	Keyghobadi *et al.* 2004
CxpGT9	$(GT)_{13}$	F: AATCTCCCCGTATAATTGTG<br>R: Ned-TATAAGACCAGTGAAGCCAG	52	Keyghobadi *et al.* 2004
CxpGT12	$(TG)_{14}$	F: AACGTGAGCGTGATTGCTC<br>R: 6-fam-CAGCTGTTGCACCAATGTC	54	Keyghobadi *et al.* 2004
CxpGT20	$(TG)_{15}$	F: CAACCGCTAAATTGCCTCA<br>R: Ned-GCAAACCCGATACCGAAT	54	Keyghobadi *et al.* 2004
CxpGT40	$(GT)_{15}$	F: CATCATCTGTCCACGATCC<br>R: Hex-TTATGCAGTTGCTGTCATATCC	52	Keyghobadi *et al.* 2004
CxpGT46	(TG)15	F: Hex-CCGACACCGTGTTCAAAGAG<br>R: TGACGACGACGGTACAAGAG	52	Keyghobadi *et al.* 2004
CxpGT51	$(TG)_4CG(TG)_{15}$	F: GAGTATCGCTCGTTGGAGATT<br>R: Hex-ACCCTCTTTTCTTTCTATGTCTGT	54	Keyghobadi *et al.* 2004
CxpGT53	$(TG)_{22}$	F: 6-fam-GTCCCGTTTGGTTGGTTG<br>R: CCATCTCCTCCTGAATCCTG	58	Keyghobadi *et al.* 2004
CxqGT4	$(GT)_{12}$	F: ATAGAACTTGTTCGCCGTCTC<br>R: 6-fam-TCTAAACACGCACCACGTACA	52	Smith *et al.* 2005
CxqGT6b	$(CA)_8$	F: CAACCAGCAAAACCCTCATC<br>R: Ned-TAGCCGGGCAGATTCATTAC	54	Smith *et al.* 2005
CxqTri4	$(TGC)_7$	F: Hex-CTAGCCCGGTATTTACAAGAAC<br>R: AACGCCAGTAGTCTCAGCAG	54	Smith *et al.* 2005

T_A: annealing temperature. Fonseca *et al.* 1998. *Molecular Ecology* **7**: 1613-1621. Keyghobadi *et al.* 2004. *Molecular Ecology Notes* **4**: 20-22. Smith *et al.* 2005. *Molecular Ecology Notes* **5**: 697–700.
